# Teaching Medical Students and Residents to Practice Trauma-Informed Care in Inpatient Psychiatric Settings

**DOI:** 10.1007/s40596-025-02280-x

**Published:** 2025-11-20

**Authors:** David S. Im, Carrie M. Tamarelli

**Affiliations:** University of Michigan Medical School, Ann Arbor, MI USA

**Keywords:** Trauma-informed care, Inpatient psychiatry, Education, Medical students, Residents

## Abstract

**Objective:**

Inpatient psychiatric units provide needed care for individuals experiencing acute mental health issues, but many individuals report negative experiences from hospitalization, and most have histories of trauma, increasing the risk of retraumatization in these settings. The promotion of trauma-informed care over the last two decades represents a step in the right direction, but specific training of medical students and residents on how to apply this to clinical practice in inpatient psychiatric settings is lacking. The authors conducted a narrative review to fill this gap in the literature.

**Methods:**

A search of the databases PubMed, Ovid MEDLINE, PsycINFO, CINAHL, and Embase was conducted in December 2024 using relevant search terms. After reviewing titles, abstracts, full-length articles, and reference lists, 49 articles were identified and reviewed.

**Results:**

The included articles represented a wide range of countries, study approaches, and participant demographics. Based on the review of the data, the authors identified 25 concrete, practical strategies that medical students and residents can be educated on to apply trauma-informed care to their clinical practice in inpatient mental health settings.

**Conclusions:**

From increasing sensitive inquiry of all patients about trauma, to engaging patients in collaborative discussion about treatments, to educating staff about the widespread impacts of trauma (including microaggressions) and how observed patient behaviors may be related to this, to seeking and advocating for training in the care of specific populations, the authors’ findings can inform the education of medical students and residents on the use of trauma-informed care in inpatient mental health settings.

Inpatient psychiatric units provide pivotal care for individuals experiencing acute mental health issues leading to concern about intentional or unintentional harm to themselves or others. However, many individuals report negative experiences being hospitalized, citing such factors as a lack of empathic connection with staff, a lack of communication from treating physicians, a non-therapeutic physical environment, and feeling little control over their treatment [1]. Moreover, most individuals admitted to inpatient psychiatric units have a history of psychological trauma such as physical, sexual, or emotional abuse [2], increasing their vulnerability to being retraumatized on the unit due to such factors as coercive interventions (like the use of seclusion or restraint) or perceived invalidating or antagonistic interactions with patients or staff.

Awareness of these issues has led to the promotion of trauma-informed care in mental health settings by organizations such as the Substance Abuse and Mental Health Services Administration (SAMHSA), the National Association of State Mental Health Program Directors (NASMHPD), and Trauma-Informed Care Resources [3]. Key principles of trauma-informed care that have been highlighted by these organizations include (1) safety; (2) trustworthiness and transparency; (3) peer support; (4) collaboration and mutuality; (5) empowerment, voice, and choice; and (6) cultural, historical, and gender issues [4].

Some authors have noted that adult inpatient mental health units tend to focus their care on risk management, illness assessment, and medical stabilization [5, 6], with a corresponding de-emphasis on therapeutic relationships and “talking” therapies with patients. These authors have reported that staff, especially those working in acute units with involuntarily hospitalized patients, appear largely preoccupied with medication and coercive activities, such as enforcing ward rules and treatment orders, undertaking close observations, and managing patient aggression and self-harm with physical interventions. This type of treatment culture has been perceived by patients and their families as controlling and unhelpful, in contrast to units emphasizing psychological support and growth, where patients and families perceive staff as more caring and supportive when they focus on therapeutic relationships and interventions to build self-determination and autonomy [7, 8].

Most literature on trauma-informed care in inpatient psychiatric settings centers on training nurses and front-line staff to reduce coercive practices like seclusion or restraint [9] and to interact with patients in a sensitive, trauma-informed manner [10]. In contrast, there is little focus on educating medical students and residents in these principles. Although effective trauma-informed care requires commitment from all organizational levels [11], specific guidance for medical learners is notably lacking. In our experience, medical students and residents rarely receive formal training in trauma-informed care, beyond education on trauma-related conditions like post-traumatic stress disorder (PTSD). However, training in PTSD diagnosis and treatment is not equivalent to being well-versed in trauma-informed care, which is essential for all inpatient mental health care, given the high prevalence of trauma among these patients [12].

We therefore conducted a narrative review of the scientific literature on the use of trauma-informed care in inpatient psychiatric settings, with a goal of identifying concrete, practical strategies medical students and residents can be educated on to practice such care in these settings. To our knowledge, this is the first attempt to synthesize the literature in order to enhance the training of medical students and residents on specific ways to practice trauma-informed care in inpatient psychiatric settings. Our hope is that the findings from this review can inform educational and lifelong learning efforts in this regard.

## Methods

**Fig. 1:**
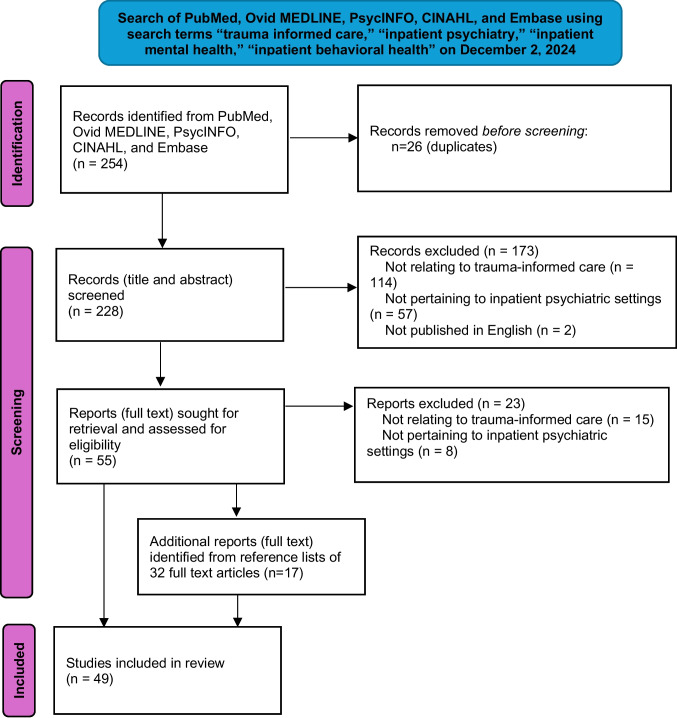
Schematic of search process for use of trauma-informed care in inpatient psychiatric settings

Figure 1 presents a schematic of the search process for our review. We searched PubMed, Ovid MEDLINE, PsycINFO, CINAHL, and Embase from inception to December 1, 2024, on December 2, 2024, using the terms “trauma informed care,” “inpatient psychiatry,” “inpatient mental health,” and “inpatient behavioral health.” Inclusion criteria were (1) articles on trauma-informed care in inpatient psychiatric settings and (2) English-language publications. An initial search with the terms “trauma informed care,” “medical students,” “residents,” “physicians,” plus the above inpatient terms revealed no citations on training on or use of trauma-informed care by medical students and residents in inpatient psychiatry. Therefore, we broadened our search to review all literature on trauma-informed care in these settings, intending to analyze included articles for implications on educating medical learners.

The initial search yielded 254 citations; after the removal of duplicates, there were 228 citations. Of these, 173 were excluded after a review of titles and abstracts (114 for not specifically relating to trauma-informed care, 57 for not pertaining to inpatient psychiatric settings, and 2 for not being published in English). Full-text articles of the remaining 55 citations were read in full; of these, 23 were excluded (15 for not specifically relating to trauma-informed care, and 8 for not pertaining to inpatient psychiatric settings). Reference lists of the remaining 32 citations were reviewed, yielding an additional 17 citations, for a total of 49 articles meeting inclusion criteria for this narrative review.

As noted above, we then carefully examined each of the reviewed articles to identify any practical implications of the findings in terms of how medical students and residents can be educated to practice trauma-informed care on inpatient psychiatric units. All included articles were reviewed and data extracted independently by each of the study’s authors (D.I. and C.T.), with differences in opinion resolved by discussion until consensus was reached.

For this review, we followed standards outlined by Sukhera [13] for narrative review reporting because of its practical, clear presentation of the essential components of a narrative review, and to ensure a deliberate, rigorous approach to using this method, including providing (1) a rationale for the narrative review, (2) clarity regarding the scope of the review and relevant definitions, (3) justification for inclusion and exclusion criteria, (4) information on reflexivity and a saturation/sufficiency statement, and (5) analysis and interpretation of the data gathered. In terms of no. 4, based on our experience as inpatient psychiatry faculty at an academic medical center, we limited our sampling to articles describing the use of trauma-informed care in inpatient psychiatric settings. Citations were assessed for inclusion in our review until saturation was reached; i.e., additional data did not lead to any new emergent themes.

## Results

Forty-nine studies were identified in our review as relevant to trauma-informed care on inpatient psychiatric units, including any implications from these studies on how medical students and residents can be educated to practice trauma-informed care in these settings [2, 3, 9–12, 14–56]. In terms of study designs, 14 studies utilized semi-structured [15, 16, 19, 21, 22, 25, 28, 35, 46, 52, 54, 55] or structured [12, 45] interviews of patients and/or staff, 10 utilized record review [9, 16, 18, 23, 24, 38, 40, 41, 45, 53], 6 employed surveys (including self-report instruments) of patients and/or staff [2, 20, 29, 37, 45, 52], 8 were opinion articles (including commentaries and editorials) [10, 11, 14, 33, 39, 44, 48, 50], 3 were descriptive articles [26, 27, 34], 4 were narrative reviews [3, 32, 47, 56], 2 were systematic reviews [30, 42], 1 was a scoping review [43], 3 were case reports [32, 49, 51], 1 used a randomized controlled design [17], and 1 used national forensic census data [36]. Of note, 3 studies used more than one type of design approach [16, 45, 52].

The included articles collectively featured participants ranging in age from 8 to 74 years, treated in inpatient psychiatric settings that included community psychiatric hospitals [2, 19, 20, 26, 29, 30, 48, 51, 52], acute psychiatric units within general hospitals [11, 12, 15, 21, 23, 25, 27, 31–35, 37, 43, 46, 47, 49, 53, 54], secure forensic psychiatric hospitals [16, 24, 28, 36, 38, 41, 55], state psychiatric hospitals [9, 10, 17, 18], and dual diagnosis inpatient psychiatric units [40]. Diagnoses, where specified, included disruptive behavior disorders [18], depression [2, 18, 39], bipolar disorder [2, 18, 36], post-traumatic stress disorder (PTSD) [37, 39], reactive attachment disorder [18], psychotic disorders [2, 18, 36], anxiety disorders [18], autism spectrum disorder [18], and intellectual disability [16, 18].

Where specified, a variety of countries were represented in terms of study settings (Table 1), with 12 studies occurring in the USA [9, 15, 17, 18, 24, 26, 35, 37, 41, 51–53]; 6 in Australia [21, 23, 25, 27, 31, 34]; 2 in England [16, 28]; 2 in Canada [20, 49]; 1 each in Finland [19], Scotland [36], the UK [38], China [2], Sweden [45], New Zealand [46], and the Netherlands [55]; and a combination of 17 Arab countries [29].

**Table 1:** Countries represented in included articles on trauma-informed care in inpatient psychiatric settings^a^

Country	Number of articles	Implications for educating medical learners in trauma-informed care (examples)
USA	12	Medical learners can be educated to practice TIC by: • viewing patients’ behavior as trauma-related [15] • asking patients how staff can support their safety on the unit [15] • fostering trust via collaborative treatment planning [15] • involving patients in development of policies [9] • debriefing after seclusion/restraint incidents [9] • inviting patients and families to construct an illness narrative [26] • considering use of positive behavior support plans to reduce use of seclusion and restraint [53]
Australia	6	Medical learners can be educated to practice TIC by: • interacting with patients in manner that acknowledges strengths and vulnerabilities and facilitates trust and sense of control [21] • asking about trauma after building rapport [23] • being aware of widespread impacts of trauma and when it may be overlooked (e.g., patients with psychosis, over 60, of opposite gender to clinician) [23] • integrating trauma histories into formulations of patient’s condition and treatment plan [23] • using evidence-based approaches for agitation [27]
England	2	Medical learners can be educated to practice TIC by: • providing trauma-specific education for staff to promote compassionate approach [16]
Canada	2	Medical learners can be educated to practice TIC by: • offering patients quiet spaces (e.g., comfort rooms) [20] • debriefing after seclusion/restraint episodes [20] • facilitating staff education in TIC principles [49] • allowing room for flexibility and adapting to individual patient’s needs [49]
Finland	1	Medical learners can be educated to practice TIC by: • Encouraging provision of meaningful activities [19] • Engaging in pre-incident planning [19] • Documenting patients’ wishes [19]
Scotland	1	Medical learners can be educated to practice TIC by: • Encouraging environments that model healthy boundaries and non-threatening interactions [36] • Providing opportunities for patients to learn and rehearse self-regulation, self-reflection, and self-correction skills [36]
UK	1	Medical learners can be educated to practice TIC by: • mindfully asking all patients about any history of trauma and accurately assessing for trauma symptoms, including patients with psychosis [38] • providing appropriate treatment for patients assessed to be experiencing trauma symptoms or meeting criteria for PTSD [38]
China	1	Medical learners can be educated to practice TIC by: • routinely screening and monitoring patients with severe mental illness for trauma [2] • employing evidence-based trauma assessment and intervention [2] • normalizing reporting and help-seeking for trauma [2]
Sweden	1	Medical learners can be educated to practice TIC by: • encouraging patients to establish and maintain healthy interpersonal relationships and engage in their psychiatric care [45]
New Zealand	1	Medical learners can be educated to practice TIC by: • encouraging patients to complete advance directives to facilitate greater choice-making [46] • inquiring of patients if they have provider gender preference [46] • offering trauma-specific psychological therapies if trained in these [46]
The Netherlands	1	Medical learners can be educated to practice TIC by: • being aware of prevalence and impact of trauma [55] • recognizing signs and symptoms of trauma (e.g., considering outbursts as result of provocation or microaggressions by others) [55] • ensuring everyone consider a patient’s experience of trauma and use safe, collaborative, empathic interactions with patients instead of rigid limit-setting and punitive consequences [55]
Combination of 17 Arab countries	1	Medical learners can be educated to practice TIC by: • identifying cultural barriers to discussion of mental health symptoms and care seeking [29] • considering ways that primary care and educational settings may increase access to mental health (including trauma-informed) care [29]

^a^Based on articles that specified this information (30 of the 48 included articles)

*PTSD*, post-traumatic stress disorder; *TIC*, trauma-informed care

While all articles addressed individuals receiving care in inpatient psychiatric settings, some focused on specific populations, such as women [21, 23, 28, 42, 43, 48], youth [12, 18, 30, 37, 44, 47, 51, 52], transgender individuals [32, 35, 39], individuals with intellectual disability [16, 28], trafficking victims [33], and military veterans [56]. Table 2 shows populations examined in the included articles.

**Table 2:** Populations examined in included articles on trauma-informed care in inpatient psychiatric settings^a^

Population	Number of articles	Implications for educating medical learners in trauma-informed care (samples)
Unspecified/ Undifferentiated	28	Medical learners can be educated to practice TIC by: • asking about trauma histories at admission [9] • operating from a strengths-based perspective to highlight coping and problem-solving abilities [14] • empowering patients with collaborative treatment planning [15] • debriefing patients after seclusion/restraint [9] • learning and using CBT, CET, DBT, and/or sensory modulation to encourage empowerment and self-efficacy [10] • inviting patients and families to construct an illness narrative [26] • using evidence-based strategies for agitation [27] • encouraging use of advance directives [46] • considering PBS plans to reduce restraint use [53]
Youth	8	Medical learners can be educated to practice TIC by: • screening youth for history of trauma [12] • personalizing care (language, documentation) [12] • having consistent way to document trauma history so youth doesn’t have to repeat to each new provider [12] • using “universal precautions” approach that ensures each patient, caregiver, and staff member is approached with trauma-informed sensitivity [12] • using seclusion/restraint data to inform practice [18] • educating staff in trauma effects and recovery principles [18, 30] • including youth and families in care planning [18, 30] • recognizing that trauma can mimic other disorders and ensuring diagnostic clarity [44] • de-prescribing when emotion dysregulation persists despite complex polypharmacy [44] • increasing multidisciplinary communication for patients requiring frequent crisis interventions [51]
Women	6	Medical learners can be educated to practice TIC by: • asking sensitively about trauma after building rapport [23, 42] • recognizing strengths and needs to foster trust and control [21, 42] • interacting in gender-sensitive manner that acknowledges unique needs (e.g., as a mother) [42] • being aware of widespread impacts of trauma and of patients who tend to not have trauma asked about (e.g., those of opposite gender to clinician) [23] • integrating trauma histories into treatment plans [23] • seeking training and supervision on how to explore and support women disclosing experiences of abuse [42]
Transgender individuals	3	Medical learners can be educated to practice TIC by: • recognizing that inpatient psychiatric units offer chance to instill hope, provide validation, and advocate for transgender patients [32] • using correct names and pronouns and ensuring team-wide confidentiality and sensitivity [32, 39] • applying gender-affirming care by using accurate identifiers and apologizing when mistakes are made [35, 39] • screening for abuse, suicidal ideation, and substance use [39]
Individuals with intellectual disability	2	Medical learners can be educated to practice TIC by: • routinely assessing for mental health conditions, including trauma and PTSD [16] • adapting evidence-based psychological treatments [16] • providing trauma-specific education for staff to promote compassionate approach [16] • talking with patients about why they are upset before moving to use of restraints [28]
Victims of trafficking	1	Medical learners can be educated to practice TIC by: • minimizing exposure to aggression [33] • providing safe spaces to discuss trafficking history [33] • ensuring access to all necessary medical care [33] • liaising with legal and immigration agencies to advocate regarding legal concerns (e.g., from being forced into illegal activities by traffickers) [33]
Military veterans	1	Medical learners can be educated to practice TIC by: • recognizing heightened aggression or retraumatization risk, particularly for female veterans or those with history of military sexual trauma [56] • helping veterans build physical and emotional safety by establishing clear roles and boundaries and prioritizing privacy, confidentiality, and mutual respect [56] • encouraging veterans to make choices to rebuild a sense of personal control [56]

^a^Based on articles that specified this information (30 of the 48 included articles)

*CBT*, cognitive-behavioral therapy; *CET*, cognitive enhancement therapy; *DBT*, dialectical behavioral therapy; *PBS*, positive behavioral support; *PTSD*, post-traumatic stress disorder; *TIC*, trauma-informed care

Gender distributions of patient respondents were specified in 22 studies [2, 9, 16, 18–24, 28, 32, 36–38, 40–42, 45, 46, 54, 55]. Of these, 13 described a greater proportion of male than female respondents (including 2 all-male studies and 1 case report of a male inpatient) [9, 16, 19, 20, 24, 32, 36, 38, 40, 41, 45, 54, 55], 7 noted a greater proportion of female than male respondents (including 3 all-female studies) [18, 21, 23, 28, 37, 42, 46], and 2 reported an equal proportion of male and female respondents [2, 22]. Two studies identified a small proportion of transgender respondents [24, 46].

Five studies [18, 24, 37, 38, 41] specified patient racial distributions (Table 3), all reporting the largest proportion as White, followed by Black, then Hispanic, with the lowest proportions among Native American, Asian-Pacific Islander, mixed, and other groups.

**Table 3:** Racial distributions of respondents in included articles on trauma-informed care in inpatient psychiatric settings^a^

Study	Racial distribution	Implications for educating medical learners in trauma-informed care (samples)
Azeem et al. (2011)^18^	63.3% White 12.6% Black 6.3% Hispanic 8.3% Other	Medical learners can be educated to practice TIC by: • encouraging leaders commit to organizational change • using seclusion/restraint data to inform practice • educating staff in neurobiological, psychological, and social effects of trauma and recovery-oriented principles (e.g., respect, dignity, self-management) • using seclusion/restraint reduction tools (such as use of comfort rooms and de-escalation techniques) • improving patient roles in safety planning and goal setting
Stinson et al. (2016)^24^	54.3% White 42.0% Black 1.6% Hispanic 2.1% Native American, Asian/Pacific Islander, or mixed race/other	Medical learners can be educated to practice TIC by: • supporting creation of safe environments that model healthy boundaries and respectful, non-threatening interactions • allowing opportunities for patients to learn and rehearse self-regulation skills, self-reflection, and self-correction
Darnell et al. (2019)^37^	73.4% White 17.0% Other or mixed race/ethnicity 4.3% Asian 2.1% Black 2.1% Latino/Latina 1.1% Pacific Islander	Medical learners can be educated to practice TIC by: • screening for trauma and trauma-related symptoms, given that avoidance is a core PTSD symptom and can lead to missed detection of trauma symptoms among adolescents with externalizing difficulties • utilizing treatments that reduce risk behaviors and prevent recurrent trauma in adolescents (e.g., transdiagnostic treatments that address comorbid externalizing and internalizing symptoms)
McKenna et al. (2019)^38^	71.6% White British 7.7% Any other White background 4.6% Black Caribbean 3.1% White and Black Caribbean 3.1% Any other Black background 2.1% Any other Asian background	Medical learners can be educated to practice TIC by: • mindfully asking all patients about any history of trauma and accurately assessing for trauma symptoms, including patients with psychosis who are at risk of not having trauma histories identified • providing appropriate treatment for patients assessed to be experiencing trauma symptoms or meeting criteria for PTSD • developing empathic, boundaried relationships with forensic inpatients
Stinson et al. (2021)^41^	56% White 40% Black 2% Hispanic 2% Mixed race/other	Medical learners can be educated to practice TIC by: • supporting training of clinical and front-line staff in TIC given high prevalence of adverse childhood experiences in forensic psychiatric inpatients

^a^Based on articles that specified this information (5 of the 48 included articles)

*PTSD*, post-traumatic stress disorder; *TIC*, trauma-informed care

Table 4 summarizes key themes based on a review of findings from the 49 reviewed studies regarding concrete, practical ways that medical students and residents can practice trauma-informed care on inpatient psychiatric units. The listed strategies are grouped into five categories corresponding to five of the six key principles of trauma-informed care articulated by SAMHSA [4]. These categories include safety (10 strategies); trustworthiness and transparency (5 strategies); collaboration and mutuality (2 strategies); empowerment, voice, and choice (3 strategies); and cultural, historical, and gender issues (4 strategies).

**Table 4:** Practical strategies for medical students and residents to practice trauma-informed care in inpatient psychiatric settings

**Safety**
1. Directly ask the patient on admission, in a mindful and sensitive manner after building rapport, about any history of past or recent trauma, and respond with compassion [2, 3, 12, 14, 23, 38, 42, 43, 48] • Keep in mind patients at risk of not having trauma histories or symptoms identified (psychotic symptoms, over 60 years old, of opposite gender to interviewer) [23, 38, 42] • Can facilitate co-creation of individualized de-escalation or safety plans with patients [9, 15, 19, 48] • Provide appropriate treatment for patients assessed to be experiencing PTSD sxs [2, 38, 46]
2. Have a consistent way to document a patient’s trauma history to other team members so that the patient does not have to repeat trauma stories to each new provider (e.g., easy-to-find location in electronic medical record) [12, 14, 52]
3. Avoid situations that may retraumatize patients (e.g., unnecessarily using seclusion/restraint or intramuscular medications, rooming trafficked/abused patients next to aggressive patients) [14, 17, 18, 33, 42, 47, 48, 51, 53, 54]
4. Seek connections between a patient’s presenting symptoms (including “disruptive” behaviors on the unit) and their trauma history, including the possibility that behavioral outbursts may be due to microaggressions by others [15, 24, 44, 47, 51, 55]
5. Encourage use of quiet spaces (e.g., comfort rooms), sensory modulation techniques, and other de-escalation approaches to reduce the need for seclusion and restraint, which can be traumatizing/re-traumatizing for patients [10, 18–20, 36, 51, 52, 56]
6. Use data (e.g., rates of seclusion/restraint, rates of intramuscular medication use) to inform practice regarding use of TIC on the unit [2, 3, 18, 30, 52]
7. Be aware of the prevalence and widespread impacts of trauma, and • Ensure everyone in the organization consider a patient’s experience of trauma and use empathic, collaborative interactions with patients instead of rigid limit-setting and punitive consequences [12, 14, 17, 18, 20, 21, 23, 30, 36, 40, 47, 48, 50, 51, 55] • Educate team members that microaggressions are more abundant than physical and sexual assaults and can traumatize patients [55]
8. Use evidence-based medication approaches for agitation or need for rapid sedation to avoid harmful effects from inappropriate prescribing [27, 42, 44]
9. For victims of trafficking, • Minimize exposure to aggressive patients to reduce risk of retraumatization [33] • Provide patients safe spaces to process details of their trafficking history [33] • Consult with medical, surgical, and gynecological colleagues to ensure access to quality health care [33] • Have the team liaise with local immigration and legal aid agencies to advocate for patients who face legal concerns (e.g,. from being forced into illegal activities by traffickers) [33]
10. For youth in inpatient psychiatric settings, • Recognize that trauma can mimic other disorders, prompting diagnostic clarity when youth do not respond to first-line treatments [44, 47, 51] • Systematically de-prescribe when emotion dysregulation persists despite complex polypharmacy [44] • Recognize the lack of strong evidence base for medications in treating PTSD in youth [44]
**Trustworthiness and transparency**
11. Integrate trauma histories into formulations of patients’ mental health condition and management plan [23, 38, 47, 50]
12. Operate from a strengths-based perspective that openly recognizes the patient’s coping and problem-solving abilities as well as vulnerabilities [14, 21, 27, 56]
13. Facilitate trust with patients by sharing information and collaboratively discussing treatment options, including medications [15, 17, 18, 21, 30, 42, 48, 54, 56]
14. Advocate for leadership to commit to organizational change to TIC by • making it a standing item in high-level meetings, allocating resources, setting clear targets, explaining the rationale for a TIC transition to staff, and conveying belief that TIC goals are achievable [11, 18, 30, 52] • supporting staff by providing training and supervision in TIC [18, 30, 31, 35, 40, 41, 43, 52] • tracking data and outcomes by regularly sharing seclusion/restraint data in grand rounds and staff meetings [2, 18, 30, 52] • aligning policy, practice, and the unit milieu with TIC principles (e.g., making the unit space safe and welcoming for patients/staff, visibly posting TIC-based mission and vision statements) [11, 30, 48, 51, 52]
15. In patients with first-episode psychosis (FEP), consider the use of care coordinators (such as an EIPS coordinator) who can facilitate trusting relationships with patients and families and advocate for providing patients choices (e.g., shared decision-making regarding medications) [54]
**Collaboration and mutuality**
16. Educate staff on the neurobiological, psychological, and social effects of trauma and on recovery-oriented principles such as patient-centered care, respect, dignity, partnership, and self-management, which can help staff more effectively interact with patients and minimize behaviors that elicit trauma-related reactions [16–18, 23, 52]
17. Allow room for flexibility and adapting to individual patients’ needs (e.g., work toward a particular discharge timeline based on a patient’s expressed preference or concerns; allow limited access to devices to allow patients to address stress-increasing work or school issues) [15, 19, 49]
**Empowerment, voice, and choice**
18. Become trained (if not already so) in CBT, CET, DBT, or sensory modulation techniques that can help encourage patient empowerment, self-control, and self-efficacy [10]
19. Make active efforts to understand the illness experience for patients and their families by inviting them to construct an illness narrative [26]
20. Encourage patients to complete psychiatric advance directives to faciltate greater choice-making [46, 54]
**Cultural, historical, and gender issues**
21. Embrace a racially and culturally responsive orientation that includes identifying cultural barriers to discussion of mental health symptoms and care seeking (e.g., consider use of interpreter services to address language barriers) and fostering support networks [12, 29, 48]
22. Recognize that inpatient psychiatric units offer a chance to provide hope, validation, and advocacy for transgender and gender non-binary patients; this includes using preferred names and pronouns in a nonjudgmental, validating manner and discussing issues regarding name and pronoun use, confidentiality, and sensitivity (e.g., reconsider gender-segregated programming) with all treatment team members [32, 35, 39]
23. Regarding female patients, • Interact with female patients in a gender-sensitive manner that facilitates choices and acknowledges their unique needs (e.g., as a mother) [42] • Increase awareness of negative attitudes toward female patients and of gender-based violence in inpatient psychiatric settings [43]
24. Ask patients if they have a treating medical learner/physician gender preference and, when feasible, try to accommodate [46]
25. Be aware of one’s own countertransference upon hearing trauma narratives and seek timely supervision to avoid overinvolvement, self-protective under-engagement, or splitting during patient care [10]

*CBT*, cognitive-behavioral therapy; *CET*, cognitive enhancement therapy; *DBT*, dialectical behavioral therapy; *EIPS*, early intervention psychosis services; *FEP*, first-episode psychosis; *PBS*, positive behavioral support; *PTSD*, post-traumatic stress disorder; *TIC*, trauma-informed care

## Discussion

As shown in Table 1, study country did not affect implications for educating medical students and residents in trauma-informed care in inpatient psychiatric settings. Similarly, implications were consistent across study populations (Table 2), though some features were more specific to certain groups. For example, for youth, learners can be instructed to involve youth and families in care planning [18, 30], recognize that trauma may mimic other disorders and re-evaluate diagnoses if initial treatments fail [44, 47], and consider de-prescribing when emotion dysregulation persists despite polypharmacy, given youths’ higher sensitivity to side effects [44].

For women, learners can be advised of the benefit of interacting with female patients in a gender-sensitive manner that acknowledges their unique needs (e.g., as a mother) [21, 42] and of seeking training and clinical supervision on how to explore and support women disclosing experiences of abuse [42].

For transgender individuals, trauma-informed training of medical learners can include discussing issues regarding name and pronoun use, confidentiality, and sensitivity among all treatment team members [32, 39], applying gender-affirming approaches to the care of transgender patients (e.g., using accurate identifiers and acknowledging and apologizing when mistakes are made) [35, 39], and asking about experiences common in transgender patients, such as abuse, suicidal ideation, and substance use [39].

For individuals with intellectual disability, medical learner training can include conducting routine structured assessments (including for trauma and PTSD) [16], offering adapted evidence-based psychological treatments [16], and talking with patients about why they are upset before moving to the use of restraints [28].

For victims of trafficking, education of medical learners can emphasize minimizing exposure to aggressive patients to reduce the risk of retraumatization [33], providing patients safe spaces to process details of their trafficking history [33], consulting with medical, surgical, and gynecological colleagues to ensure access to quality health care [33], and liaising with local agencies to advocate for patients facing legal concerns (e.g., from being forced into illegal activities by traffickers) [33].

Learners should be aware that veterans’ cumulative trauma may result in aggression from being on locked inpatient psychiatric units, increasing the risk of retraumatization, especially for female veterans and those with a history of military sexual trauma [56]. Learners can be educated that a trauma-informed approach here includes promoting physical and emotional safety by clarifying roles and boundaries, respecting privacy and confidentiality, and encouraging veterans to make choices to rebuild a sense of personal control [56].

As shown in Table 3, only 5 of the 48 included articles reported respondents’ racial distribution, but no notable differences emerged in recommendations for educating medical learners in inpatient trauma-informed care based on race. For instance, the two studies with more Black respondents (40–42%) [24, 41] offered similar guidance—emphasizing interpersonal approaches that promote safety, collaboration, and empowerment—as the predominantly White studies.

For recommendation #1 (Table 4)—directly asking about trauma on admission—medical students and residents should be aware of patients at risk of having trauma histories overlooked, such as those with psychosis, over 60, or of a different gender than the interviewer [23, 38, 42]. Research shows that patients with diagnoses like schizophrenia or schizoaffective disorder are less likely to have PTSD properly documented [57], partly due to assumptions that trauma reports in psychosis are fabricated [23]. However, trauma reports from individuals with psychosis are typically valid and stable over time [58]. Clinician retraining—increasing awareness of trauma, developing sensitive history-taking skills, and building rapport—can improve detection, with even brief training significantly increasing the identification of patient trauma [23]. Learners should focus on understanding “What has happened to this person?” rather than “What is wrong with the person?” [14].

Although most inpatient psychiatric medical students and residents lack official leadership roles, they may informally lead multidisciplinary clinical teams. For strategy #7 (Table 4), they can model “safe, collaborative, empathic interactions with patients instead of rigid limit-setting and punitive consequences” [55]; provide feedback on staff-patient interactions; and share these principles in team discussions, especially about challenging patients. They should also learn to recognize microaggressions—“intentional or unintentional hostile, derogatory, or negative slights and insults so pervasive in daily interactions that their potential impact is often not recognized” [55]—and educate staff on their potentially traumatizing effects.

Recommendation #14 (Table 4)—to facilitate trust with inpatients by sharing information and collaboratively discussing treatment options, including medications—should be fairly easy to teach most inpatient psychiatric medical students and residents, as these groups are accustomed to having informed consent discussions with patients regarding medications initiated during the course of psychiatric hospitalization. Learners can be advised that offering not only information on proposed medication treatments, but also choices between two or more medication options or between evidence-based medication and non-medication interventions for a given condition enhances patients’ sense of agency, control, empowerment, collaboration, and mutuality in the treatment relationship.

While inpatient psychiatric medical students and residents may not be in executive leadership positions, they can be encouraged to advocate for resources to implement such recommendations as “supporting staff by providing training and supervision in trauma-informed care” [30] and “aligning policy, practice, and the unit milieu with trauma-informed care principles (e.g., making the unit space safe and welcoming for patients/staff, using trauma-informed care-based mission and vision statements and posting them visibly)” (strategy 15, Table 4) [30].

It is also reasonable to instruct medical students and residents on recommendation #17 (Table 4): educating staff about the neurobiological, psychological, and social impacts of trauma, as well as recovery-oriented principles like respect, dignity, partnership, and self-management. Medical students and residents are already accustomed to presenting to multidisciplinary audiences. In our experience, nursing staff appreciate in-services on patient care topics given by medical learners and attendings. Learners can use online resources, including Horowitz et al.’s [10] overview of trauma effects and recovery principles, to support staff education efforts.

Finally, some studies recommended that organizations hire consultants to train faculty, staff, and administrators in trauma-informed care (e.g., The Sanctuary Institute) [26] and related topics, such as gender-affirming care for transgender individuals [35, 39] and supporting women who disclose abuse [42]. Inpatient psychiatric medical students and residents, through informal leadership roles, can advocate for funding such trainings to enhance multidisciplinary awareness of trauma-informed practices that promote safety, trust, peer support, collaboration, and empowerment for specific populations.

The following clinically illustrative vignettes can be used to specifically teach medical students and residents how to apply trauma-informed care in inpatient psychiatric practice. These are composite case examples not based on any individual patients but featuring common themes we have observed in our clinical work in an academic hospital affiliated with a large public university.

### Illustrative Vignette #1

Jane is a 28-year-old woman admitted to the inpatient psychiatric unit from the emergency room after police find her outside a local supermarket shouting obscenities at customers entering and exiting the store and eventually throwing a rock through a store window. After multiple attempts to verbally redirect her, police take her to the local emergency room. She is highly combative en route, screaming and attempting to kick out the police car window. Because of concern about her level of agitation, which continues in the emergency room, upon admission she is placed in a secure portion of the inpatient unit for closer observation. You are the psychiatry resident assigned to take care of her on the unit, and the admission interview proceeds as follows:

You: “So can you tell me why you were brought to the hospital?”

Jane (with much trepidation): “They forced me to come here! They’re all in on it, the store owners, the customers, the police, everyone! Being paid off by the same criminals who bribed the store to put poison in all of their products so that they can produce more psychopaths who go around raping women and getting away with it, and selling drugs and using the money to pay off people like you who don’t give a d--- about people like me!”

You: “If I can backtrack – according to the physician who talked to you in the ER, it sounds like the police brought you here because you were outside a store yelling at people and threw a rock through one of the windows?”

Jane: (angrily): “I was trying to stop anyone else from being poisoned by a business that promotes evil, pure evil! I thought if I could get the police’s attention, they would listen and stop all of this… August fourteenth, two-zero-one corner of Main and Maddox, three-thirty in the morning, I told them over and over again, but no one would listen… that’s how I know they’re in on it too!”

You: “So – how did you come to conclude that this store has any connection to these men doing all these horrible things?”

Jane (increasingly frustrated): “Haven’t you heard a single word I’ve said?! How come everyone I talk to defends the store, but once a woman talks about being violated, they look the other way! I’m done talking to you, you’re no different than the rest of them!”

You attempt to ask Jane a few more questions, but she becomes increasingly agitated, so you conclude the interview. You document in your admission note that while the interview was limited due to her agitation at the time, Jane appeared to be experiencing an acute psychosis, and that olanzapine would be initiated to address this, which Jane had the right to refuse until a court order could be obtained. In the ensuing days, Jane refuses to take the prescribed olanzapine, expressing frustration that none of the inpatient providers had informed her about a recommendation to start this medication or about details such as its purpose, dosage, side effects, and long-term risks.

Learners can be informed that this vignette illustrates a common scenario in inpatient psychiatric settings in which, while interviewing individuals presenting with acute agitation and psychosis, medical students and residents may initially focus on trying to make sense of patients’ accounts of what led to their hospitalization, perhaps (not unreasonably) inquiring into illogical or delusional thinking, but neglecting to inquire further into possible trauma experienced by the patient. In this case, per strategy 1 in Table 4, instead of questioning how Jane connected the store to the alleged perpetrators she was referencing, a trauma-informed response to Jane’s initial statements of concern might consist of, “You mentioned August fourteenth, which sounds like a significant date for you…what happened on that date?” If Jane proceeds to disclose that she was sexually assaulted on that date, it would be appropriate to empathically listen and clarify if Jane has sought or received any kind of medical or psychiatric attention since the alleged incident and about the safety of her current living situation. A trauma-informed approach would also entail, after establishing a kind, empathic, nonjudgmental rapport with Jane, validating her distressed emotional state and conveying that the inpatient team is committed to working together with her as a key collaborator in figuring out how to best help her during this stressful time (strategy 13, Table 4). This teamwork would include directly discussing diagnostic impressions with Jane, and any proposed treatment options, including medication options. For example, the medical student or resident could comment (after providing Jane ample time to express her concerns and an opportunity to disclose any past or recent trauma), “I’m so sorry to hear about all of these very stressful things you’ve been experiencing and expressing concern about. We know that people who have experienced traumatic events – like you have – can have all kinds of reactions to those events. In some cases the trauma affects their brain to the point of influencing how they perceive things and people in the world around them, so that they sincerely perceive people or groups of people as having harmful intent even when they don’t. I would like to help you better discern these perceptions so that you can move forward and achieve your goals in life without such thoughts and feelings hindering you. Does that makes sense? If you agree, could we talk about some possible ways to do this?”

### Illustrative Vignette #2

Chris is a 23-year-old transgender male (natally female) who was admitted because of worsening depressive symptoms and suicidal ideation over the last 3 weeks in the context of stressful family dynamics and a falling out with online friends, neither of which he wished to discuss. On interview, he appears to have left periorbital bruising, is guarded, and makes poor eye contact (mainly looking downward). He endorses the aforementioned symptoms, declines to allow the team to reach out to his family members for purposes of care/discharge planning, and expresses frustration about a number of issues, including his hospital identification wristband displaying his birth name (Christina) and birth gender (female), his medical record similarly referencing primarily his birth name and gender, and “being locked in a place where I don’t have my phone, can’t use my laptop, and no one here gets me…and it’s not subtle.” During treatment team meetings (and in direct interactions with Chris), multiple team members refer to him with female pronouns without self-correcting despite acknowledging him as a transgender male when introducing his case. While rounding, you (the resident) and the medical student overhear two staff, within earshot of patients, conversing with each other about Chris during shift-to-shift signout; one staff member comments, “Ok, the next patient, Christina – or excuse me, CHRIS, ‘cause I guess she’s a ‘he,’ or ‘he’ is really a ‘she,’ or whatever (rolling his eyes and with a sarcastic tone)…has been pretty demanding about getting her phone and computer…”

Medical students and residents can be advised that this vignette illustrates several issues commonly experienced by transgender (and gender non-binary) individuals, including higher rates of depression and suicidality related to experiences of discrimination, rejection, and violence; organizational insensitivity to Chris’ transgender status (as evidenced by featuring his birth name and gender on his hospital wristband and in the medical record); insensitivity of unit team members to Chris’ gender identity (as reflected by the use of inappropriate pronouns during team meetings and while interacting directly with him); and microaggressions by staff based on his transgender status. As noted by Walton and Baker [39], experiences of insensitivity and outright discrimination by health care professionals have caused barriers to help-seeking for transgender individuals. Per strategy 7 in Table 4, the medical student and resident in this example can be encouraged to speak with unit staff about the damaging impact of microaggressive comments and behaviors on transgender and other individuals, and to educate them regarding the abundant experiences of discrimination, rejection, and violence faced by these individuals. They can also be advised to educate staff on how the inpatient unit has the potential to provide transgender individuals hope, validation, and advocacy through such approaches as described earlier (Table 2 and strategy 22, Table 4).

While we have emphasized the role of medical students and residents in educating nursing and unit staff about trauma-informed care, the exchange is mutually beneficial. Nursing staff can teach medical learners about trauma-informed care through their own training, experience, and example, as they spend significantly more time with psychiatric inpatients and are often the first point of contact. By checking in with the patient’s assigned nurse before each visit, medical learners can gain insights into effective ways to connect with patients and convey safety, trust, collaboration, empowerment, and cultural sensitivity. Additionally, nurses with expertise in trauma-informed care can provide valuable didactic presentations to medical learners and drive programs to bring trauma-informed care and educational initiatives to interdisciplinary settings, as in some of the studies we reviewed [25–27].

This review included numerous studies from various countries, encompassing participants with diverse ages, psychiatric diagnoses, racial backgrounds, and inpatient settings. It is, to our knowledge, the first to identify practical strategies for educating medical students and residents in trauma-informed care in inpatient psychiatry. However, several limitations exist. First, the diverse methodologies of the 49 reviewed articles limit our ability to draw strong conclusions about trauma-informed care’s effectiveness due to a lack of standardized analyses. Nonetheless, many qualitative studies support benefits such as reduced use of coercive measures (e.g., seclusion, restraint) and improved patient experience. Second, of 21 studies reporting gender, only 2 included data on transgender participants; it is unclear if this reflects actual prevalence or inadequate inquiry. Given higher rates of depression, suicidality, and trauma among transgender individuals [39], future studies should include greater gender diversity. Third, while studies included multiple countries, many regions were absent, restricting global generalizability. Finally, among studies specifying race, samples were mostly White, with fewer Black and minority participants. Since racial bias can impact care—with one-third of African Americans reporting discrimination in clinical settings [59]—future studies should ensure greater racial representation.

In conclusion, inpatient psychiatric units have an important role in providing needed care for individuals experiencing acute mental health issues. However, many individuals report negative experiences being hospitalized. Most of these individuals have a history of past trauma, increasing their risk of being retraumatized in the inpatient mental health setting. The promotion of trauma-informed care at all organizational levels by SAMHSA, NASMHPD, and other entities represents a step in the right direction, but specific guidance on how medical students and residents can be educated to practice trauma-informed care on inpatient psychiatry units is lacking. This narrative review aims to fill this gap in the literature by identifying concrete, practical strategies that these groups can be educated on to apply trauma-informed care to their clinical work in inpatient psychiatric settings. From increasing mindful, sensitive inquiry about past trauma of all patients (including those experiencing psychosis, over 60, and of an opposite gender to the provider), to inviting patients to engage in collaborative discussion about medication and other treatments and sharing information that will help them make informed choices, to educating staff about the widespread impacts of trauma (including microaggressions) and how observed patient behaviors may be related to this, to seeking and advocating for training in the care of specific populations, we hope that the findings from this review can inform the education of medical students and residents, and even lifelong learning of faculty, on the use of trauma-informed care in inpatient mental health settings.

## Data Availability

No datasets were generated or analyzed during the current study.
